# Supplementary material for: Isolation, Identification, and Biocontrol Mechanisms of Endophytic Burkholderia arboris DHR18 from Rubber Tree against Red Root Rot Disease
Source: Microorganisms. 2024 Aug 29;12(9):1793. doi: 10.3390/microorganisms12091793 (PMC11433949; doi:10.3390/microorganisms12091793)
Supplement: Supplementary file 1 [file microorganisms-12-01793-s001.zip › microorganisms-3114724-supplementary.pdf]

Table S1. Genome feature of strain DHR18.

Type	Number	Total_len	Average_len	Percentage of genome (%)
Gene	7,597	7,386,926	972	87.72
CDS	7,450	7,345,767	986	87.23
tRNA	73	5,772	79	0.07
23S rRNA	6	17,274	2879	0.21
16S rRNA	6	9,174	1529	0.11
5S rRNA	5	535	107	0.01
tm RNA	1	370	370	0.00

Table S2. The plant growth promotion-associated genes in strain DHR18

SeqID	KO	Gene	Function	Potential application
ctg_04905	K01695	*trp*A	tryptophan synthase alpha chain	Plant growth regulator
ctg_05505	K00128	*aldh*	aldehyde dehydrogenase (NAD+)	Tryptophan metabolism
ctg_03473	K00128	*aldh*	aldehyde dehydrogenase (NAD+)	Tryptophan metabolism
ctg_07039	K00128	*aldh*	aldehyde dehydrogenase (NAD+)	Tryptophan metabolism
ctg_04907	K01696	*trp*B	tryptophan synthase beta chain	Plant growth regulator
ctg_00484	K01609	*trp*C	indole-3-glycerol phosphate synthase	Plant growth regulator
ctg_02598	K04751	*gln*B	regulating nitrogen utilization	Biofertilizer
ctg_03090	K04751	*gln*B	regulating nitrogen utilization	Biofertilizer
ctg_03011	K02806	*pts*N	regulating nitrogen utilization	Biofertilizer
ctg_01930	K03366	*bud*A, *bud*C	plant defense	Plant resistant elicitor
ctg_02269	K04488	*nif*U	nitrogen fixation	Biofertilizer
ctg_06505	K15790	*nif*Q	nitrogen fixation	Biofertilizer

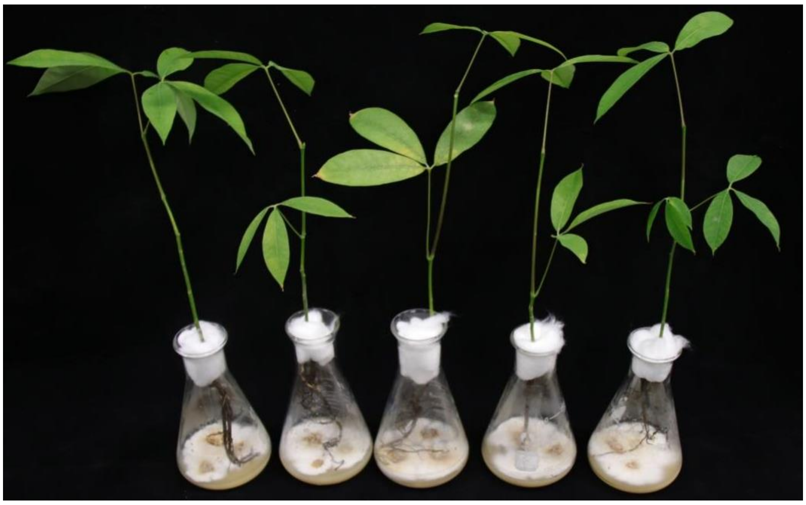

Figure S1. Schematic diagram of Ganoderma pseudoferreum inoculation on rubber tree

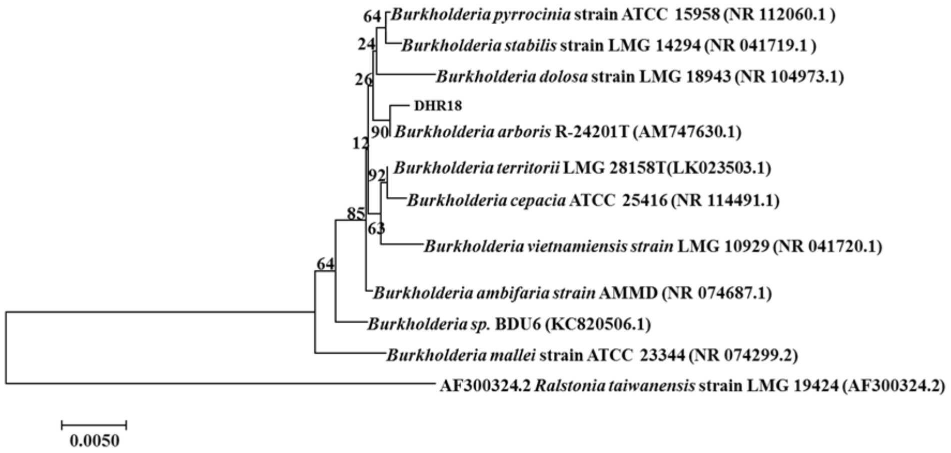

Figure S2. Phylogenetic trees based on the 16S rDNA sequence of DHR18 and their homologous sequences. *Ralstonia taiwanensis* strain LMG 19424 (AF300324.2) was chosen as the out-group.

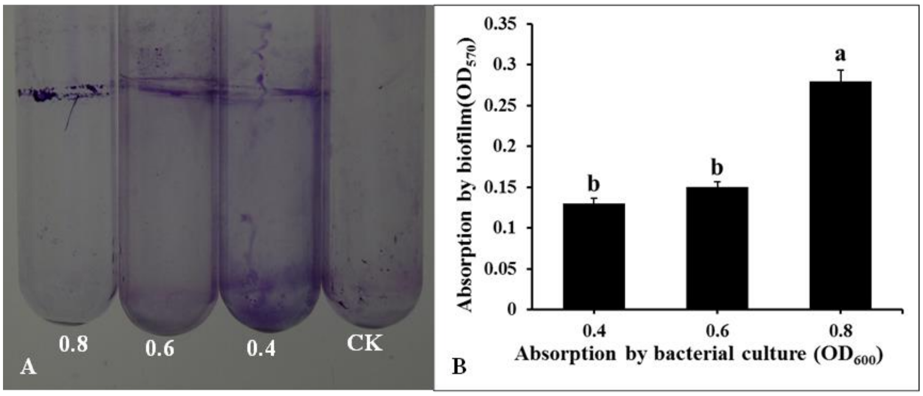

Figure S3. Biofilm formation. Biofilms developing on glass tubes were stained with 0.1% crystal violet (**A**). The mean amount of biofilm produced by different concentrations culture (OD_{600} = 0.4, 0.6, 0.8) (**B**). Bars indicate the standard error of the mean. Columns marked with the same letter are not significantly different at $p \leq 0.05$, considering Duncan’s Multiple Range Test.

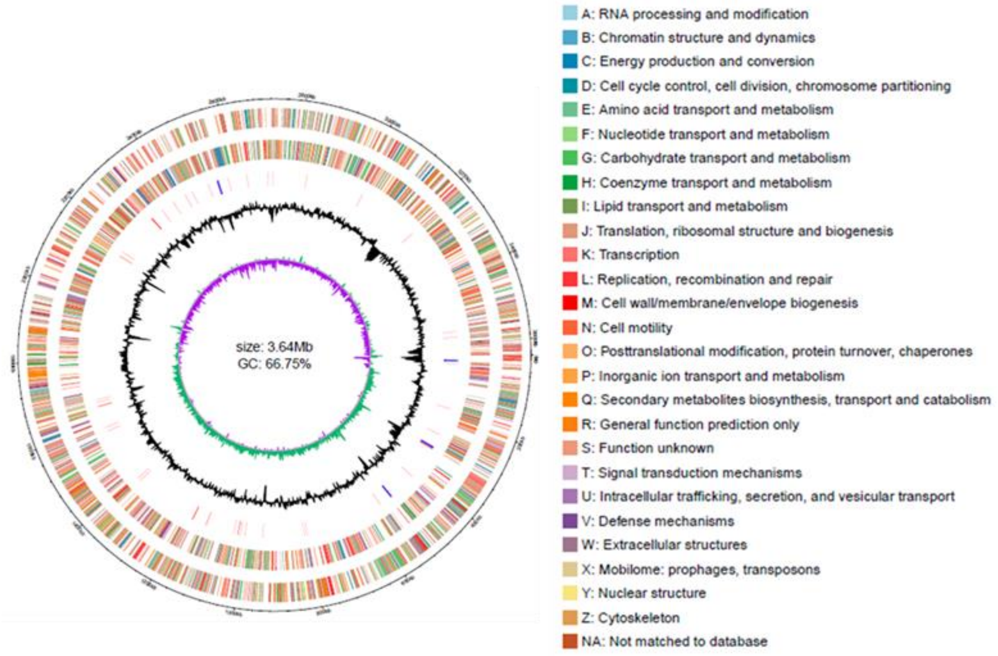

Figure S4. Circular map of strain DHR18 genome

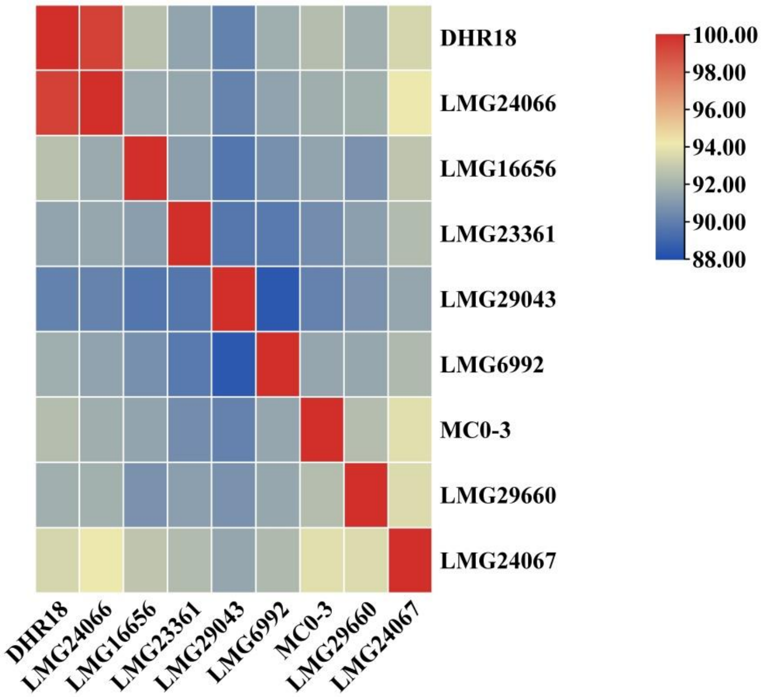

Figure S5. Average Nucleotide Identity (ANI, %) of DHR18 and other type strains. Genome comparison between DHR18 and 8 homologous bacterial strains. LMG24066: *Burkholderia arboris*; LMG16656: *Burkholderia cenocepacia*; LMG23361: *Burkholderia contaminans*; LMG29043: *Burkholderia diffusa*; LMG6992: *Burkholderia lata*; MC0-3: *Burkholderia orbicola*; LMG29660: *Burkholderia puraquae*; LMG24067: *Burkholderia seminalis*.

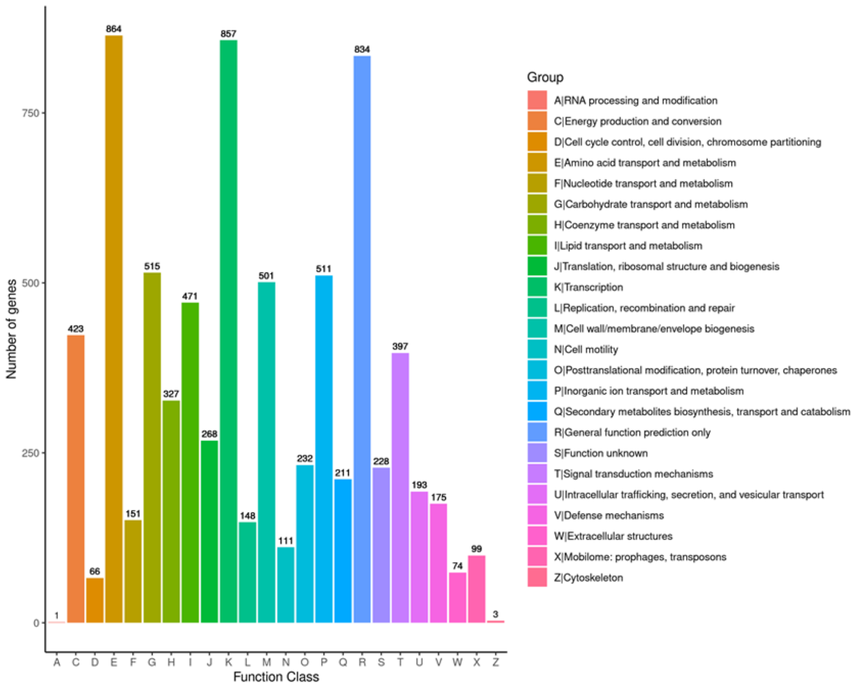

Figure S6. COG annotation of the strain DHR18 genome

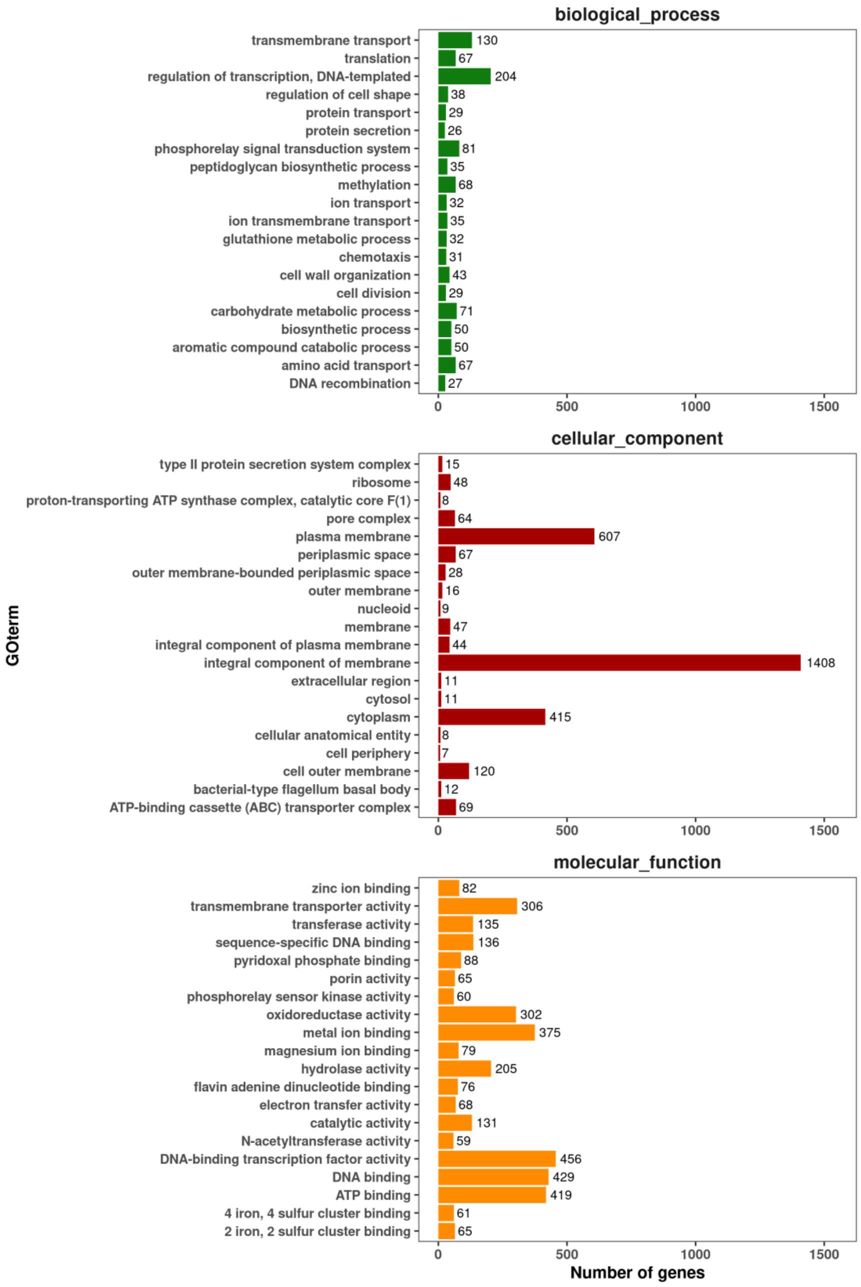

Figure S7. GO annotation of the strain DHR18 genome

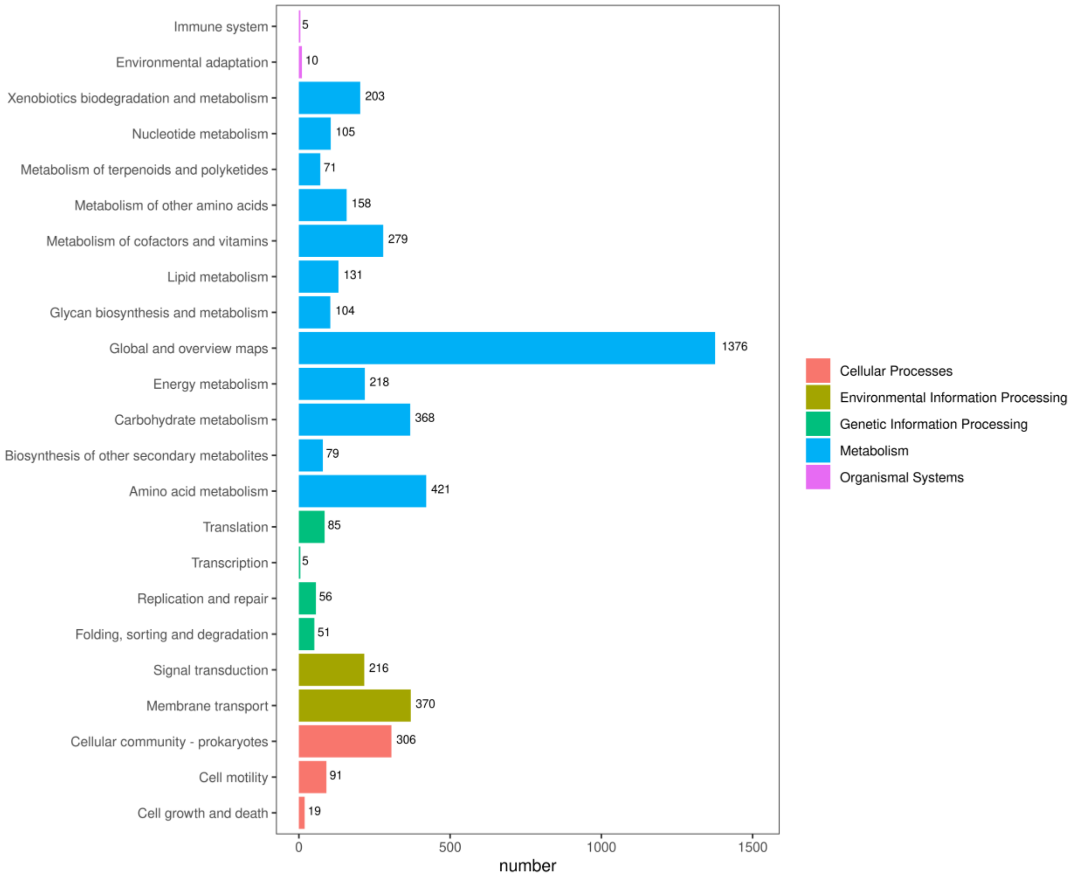

Figure S8. KEGG passageway annotation of the strain DHR18 genome

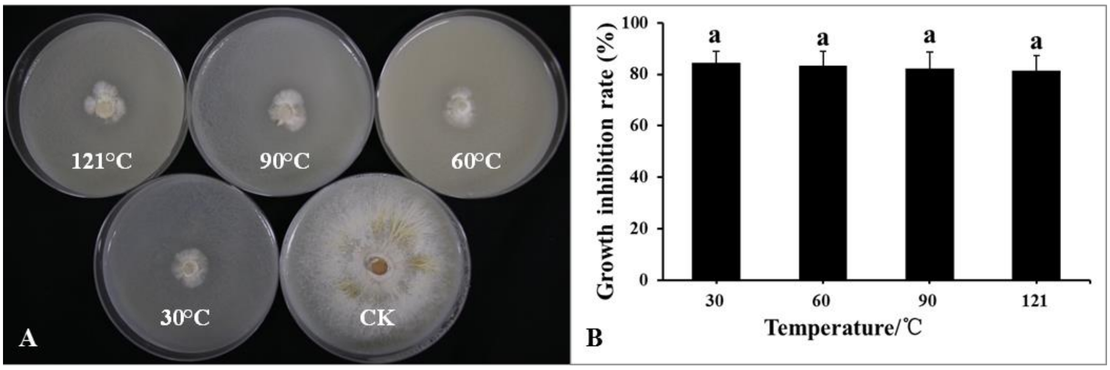

Figure S9. Effect of temperature on antibacterial activity of DHR18 **(A,B)**. Bars indicate the standard error of the mean. Columns marked with the same letter are not significantly different at $p \leq 0.05$, considering Duncan's Multiple Range Test (**B**).
